# Professor A. Snowden Piggot

**Published:** 1869-03

**Authors:** 


### EDITORIAL DEPARTMENT.

It is with the deepest sorrow we have to announce the death of our friend, colleague and associate editor Professor A. Snowden Piggot, M. D. which occurred on Saturday morning, February 13th, at the residence of Major Boggs in Spottsylvania County, Virginia.

Professor Piggot left his home in Baltimore, accompanied by his young son, on the afternoon of the 11th of February, for the purpose of visiting the mica mines in Spottsylvania County near the borders of Louisa County, in the discharge of professional engagements, expecting to return in a few days. He arrived at his destination, and after inspecting the mines returned to the residence of Major Boggs, and while seated at the table was attacked with paralysis. He was in the act of waiting upon a lady at his side, when he suddenly found himself powerless, and exclaiming that his sight was gone, the full power of the attack was manifested and he became helpless. This occurred about 7 o'clock on the evening of the 12th inst and he remained unconscious from this time until his death, about 4 o'clock the next morning.

Professor Piggot had been in failing health for nearly a year past, last summer having had quite a severe attack of heart disease which greatly enfeebled him. He had so far recovered, however, as to be able, during the present session of the Baltimore College of Dental Surgery, to faithfully and earnestly perform the duties devolving upon him as Professor of Chemistry, and greatly endeared himself to the students of the institution by his amiable and cordial manners, and his efforts to instruct them by the scientific attainments for which he had become so distinguished.

Professor Piggot graduated at the University of Maryland School of Medicine, and was for some years, professor of Anatomy and Physiology in the Medical Department of the old Washington University. On the death of Prof. Washington R. Handy, in 1858, Professor Piggot was appointed to the chair of Anatomy and Physiology in the Baltimore College of Dental Surgery, which position he held until the breaking out of the late war, when he removed with his family to the South. Being made a full surgeon with the rank of major in the Confederate service, he filled the position of superintendent of the laboratory for the manufacture of drugs, near Lincolnton, on the

Catawba river, in North Carolina. Prior to the war he had been in the habit of spending the summer months in North Carolina, devoting his time to developing the mines and minerals of this State. After the close of the war Professor PIGGOT returned to his home in Baltimore, and was appointed to the chair of Chemistry in the Baltimore College of Dental Surgery, which he occupied until his death. He was the author of a "Dental Chemistry," a "Treatise on Mines," and several standard scientific works, besides being a contributor to some of best magazine literature of this country, his articles being read with no ordinary interest. He also had in course of preparation for the press a new work on "Dental Chemistry," when he was suddenly called from labor to reward in a better world; we hope this work was so far advanced that it may yet be published. As a writer Professor PIGGOT was clear, concise, original, and his words were often eminently distinguished for their grace of expression. In all questions of scientific inquiry, he was particularly interested, and his great skill in his profession of analytical chemist, caused his opinions to be eagerly desired in many practical enterprises. As a conversationalist he was superior, being perfectly familiar with every subject which might be brought up for discussion.

In the social circle he always appeared to great advantage, and nowhere was he found more tender and uniformly courteous than in his own home. Understanding the proprieties of life so well, he bore himself in society with the self-poise of a perfect gentleman. It may be truly said of him that no father's heart was more gentle, no husband more devoted, ever solicitous for the welfare of his children, and leaving nothing in his power undone which would enhance the comfort and enjoyment of her whom he had chosen as his companion through life. In this his character was beautiful, and his loss has left a void in the home circle which can never be filled. In the death of Professor A. SNOWDEN PIGGOT, society has lost one of its most highly cultured members.

*The Funeral Services.* The remains reached Baltimore on Tuesday evening, Feb. 16th, in charge of Dr. H. M. Grant, Demonstrator of Operative Dentistry in the Baltimore College of Dental Surgery, and Dr. H. H. Keech an intimate friend of the family, both of whom had gone to Virginia for the purpose of bringing the body home for interment. Quite a number of the personal friends of Professor PIGGOT were at the depot to meet the remains on the arrival of the train.

The funeral took place on Wednesday, Feb. 17th from Grace Church, where there was a large attendance of the friends of the deceased and members of the congregation. The remains were taken to the church about noon and placed before the altar.

The officiating ministers were the Rev. Dr. Geo. Leeds, pastor of Grace Church, Rev. Geo. A. Leakin, Rev. A. P. Stryker, Rev. C. M. Calaway and Rev. Mr. Stearns, the latter an intimate friend of the deceased. The impressive services of the Episcopal Church for the burial of the dead were proceeded with, the choir and organ being heard in their sacred melodies at the appropriate intervals.

At the conclusion of the services at the church a large number of mourning friends followed the remains to Greenmount Cemetery, the following gentlemen acting as pall bearers: Professors Thomas E. Bond, Philip H. Austen, F. J. S. Gorgas, of the Baltimore College of Dental Surgery, and Dr. H. H. Keech, H. Clay Dallam, Esq., General J. Bankhead Magruder, Col. Chas. Marshall, Col. R. Snowden Andrews and Maj. Allen B. Magruder. The students of the Baltimore College of Dental Surgery under Professors Noel and Howard, and Drs. Grant and Waters, and the members of Monumental Lodge of Odd Fellows, walked in procession.

On the casket containing the remains was a silver plate bearing the inscription, "Dr. A. Snowden Piggot, died Feb. 13, 1869, in the 49th year of his age."

On the top of the casket were placed many emblems of affection, composed of rare and beautiful flowers sent as tributes of affection from the students to whom he was so greatly endeared.

At the Cemetery the services were concluded by the Revs. Messrs. Leeds and Stearns, the choir of Grace Church also participating.

THE FACULTY OF THE BALTIMORE COLLEGE OF DENTAL SURGERY, at a special meeting convened on the 15th of February, passed the following resolutions:

*Whereas,* It has pleased Almighty God to remove from our midst, in a sudden and unexpected manner, A. Snowden Piggot, M. D., Professor of Chemistry in the Baltimore College of Dental Surgery; it is, therefore,

*Resolved,* That this Faculty has learned with sincere sorrow of the death of one who for so many years has been their associate.

*Resolved,* That while we submit as becomes us to the will of the Supreme Being, we deeply deplore the great loss we have sustained in the death of our highly esteemed friend and colleague.

*Resolved,* That we shall always retain the warmest recollections of his many excellencies as a Christian, gentleman and scholar, and cherish his memory with affectionate regard.

*Resolved,* That we tender our deepest sympathy to the family and relatives of Professor Piggot for their great and irreparable loss.

*Resolved,* That the Chemical Hall of our College be draped with appropriate symbols of mourning, as a mark of our respect for the deceased, and that a copy of these resolutions be transmitted to his family.

F. J. S. GORGAS, M. D., Dean of the Faculty.

The students of the Baltimore College of Dental Surgery also called a meeting and passed the following resolutions:

WHEREAS IT HAS PLEASED AN ALLWISE PROVIDENCE to remove from his sphere of usefulness our much beloved Professor A. SNOWDEN PIGGOT, a special meeting of the STUDENTS OF THE BALTIMORE COLLEGE OF DENTAL SURGERY has been called and a committee appointed to draft suitable resolutions. The following were presented and unanimously adopted:

*Be it Resolved,*

I. That we bow with becoming humility to this stern dispensation of Almighty God, whereby Science has lost one of her most devoted followers, Society a brilliant ornament, the Medical Fraternity a prototype, and the Baltimore College of Dental Surgery one of its ablest Professors.

II. That from a sincere desire of showing every mark of respect to the memory of our deceased friend, we will attend in a body the burial service of his remains, and wear the usual badge of mourning for thirty days.

III. That we earnestly condole with the family of the deceased in their irreparable bereavement, and extend to them our heartfelt sympathy.

IV. That a copy of these resolutions be communicated to the family of the deceased, to each of the Dental Faculty, and published in the American Journal of Dental Science and Daily Sun.

Committee.

| | |
|---|---|
| T. C. EDWARDS, | GEORGE KEESEE, |
| J. B. HODGKIN, | HARRY G. ULRICH, |
| G. H. WINKLER, | T. B. YANCY, |
| J. W. PIPKIN, | B. W. SCOTT, |
| T. M. HOWARD, | JOHN C. STORY, Chairman. |

Baltimore, Md., Feb. 16th, 1869.

AT A MEETING OF THE "MARYLAND ACADEMY OF SCIENCES," held on the 15th of FEBRUARY, 1869, the following resolutions were unanimously adopted:

Whereas, The "Maryland Academy of Sciences" has received the painful intelligence of the sudden and unlooked for death of our friend and colleague, Dr. A. Snowden Piggot, therefore,

*Resolved,* That we have heard with the deepest regret of the untimely death of our fellow-member Doctor A. Snowden Piggot, in the fulness of his usefulness and professional reputation, and that we heartily deplore the loss of one so eminent in the special branch of science to which he had devoted his talents and of which he had become so distinguished an ornament.

*Resolved,* That by the death of Dr. Piggot the cause of science has lost one of its most devoted students, our community has been deprived of the valuable professional services of one of its most useful citizens, and our own Association the "Maryland Academy of Sciences," of the wise and energetic counsels of one of its most esteemed members.

*Resolved,* That we shall always cherish, in greatful recollection, the genial temper, the amiable and considerate character, the cordial manners and prudent scientific zeal of our late associate, Dr. A. Snowden Piggot.

*Resolved,* That we deeply sympathize with the afflicted family of our departed friend in their sorrowful bereavement, and that we tender to them the earnest expression of our condolence and profound respect.

*Resolved,* That the members of the Academy attend the funeral of our late colleague.

*Resolved,* That the Corresponding Secretary send a copy of these resolutions to the family of the deceased.

PHILIP T. TYSON, President.

